# Supplementary material for: Complex‐centric proteome profiling by SEC‐SWATH‐MS
Source: Mol Syst Biol. 2019 Jan 14;15(1):e8438. doi: 10.15252/msb.20188438 (PMC6346213; doi:10.15252/msb.20188438)
Supplement: Supplementary file 8 — Dataset EV7 [file MSB-15-e8438-s008.zip › feature_plots_string/P17987.pdf]

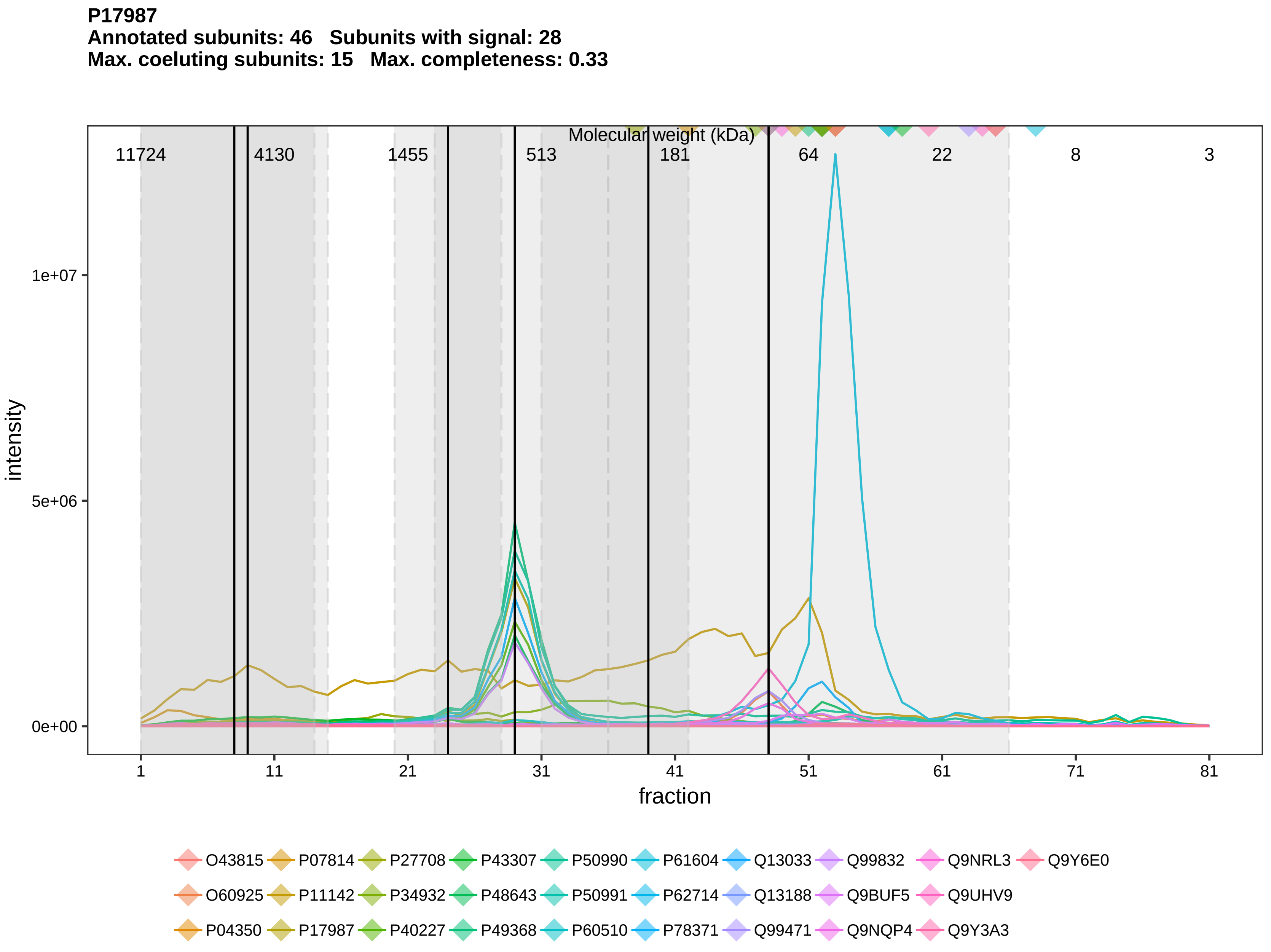
P17987
Annotated subunits: 46   Subunits with signal: 28
Max. coeluting subunits: 15   Max. completeness: 0.33
Molecular weight (kDa)
11724
4130
1455
513
181
64
22
8
3
intensity
1e+07
5e+06
0e+00
fraction
1
11
21
31
41
51
61
71
81
O43815
P07814
P27708
P43307
P50990
P61604
Q13033
Q99832
Q9NRL3
Q9Y6E0
O60925
P11142
P34932
P48643
P50991
P62714
Q13188
Q9BUF5
Q9UHV9
P04350
P17987
P40227
P49368
P60510
P78371
Q99471
Q9NQP4
Q9Y3A3
